# Functional Characterization of a Bidirectional Plant Promoter from Cotton Leaf Curl Burewala Virus Using an *Agrobacterium*-Mediated Transient Assay

**DOI:** 10.3390/v6010223

**Published:** 2014-01-14

**Authors:** Muhammad Aleem Ashraf, Ahmad Ali Shahid, Abdul Qayyum Rao, Kamran Shehzad Bajwa, Tayyab Husnain

**Affiliations:** Plant Biotechnology Laboratory, Centre of Excellence in Molecular Biology (CEMB), University of the Punjab, Lahore 53700, Pakistan; E-Mails: ahmadali.shahid@gmail.com (A.A.S); qayyumabdul77@yahoo.com (A.Q.R); kamrancembian@yahoo.com (K.S.B); tayyabhusnain@yahoo.com (T.H.)

**Keywords:** cotton leaf curl Burewala virus, agro-infiltration, bidirectional promoter, *cis*-regulatory elements, geminivirus

## Abstract

The C1 promoter expressing the AC1 gene, and V1 promoter expressing the AV1 gene are located in opposite orientations in the large intergenic region of the Cotton leaf curl Burewala virus (CLCuBuV) genome. Agro-infiltration was used to transiently express putative promoter constructs in *Nicotiana tabacum* and *Gossypium hirsutum* leaves, which was monitored by a GUS reporter gene, and revealed that the bidirectional promoter of CLCuBuV transcriptionally regulates both the AC1 and AV1 genes. The CLCuBuV C1 gene promoter showed a strong, consistent transient expression of the reporter gene (GUS) in *N. tabacum* and *G. hirsutum* leaves and exhibited GUS activity two- to three-fold higher than the CaMV 35S promoter. The CLCuBuV bidirectional genepromoter is a nearly constitutive promoter that contains basic conserved elements. Many *cis*-regulatory elements (CREs) were also analyzed within the bidirectional plant promoters of CLCuBuV and closely related geminiviruses, which may be helpful in understanding the transcriptional regulation of both the virus and host plant.

## 1. Introduction

Geminiviruses are plant pathogens with small, circular single stranded (ss) DNA genomes of 2.5–3.0 kb that are encapsidated in characteristic twin quasi-icosahedral particles. These viruses are differentiated into four genera (*Topocuvirus*, *Curtovirus*, *Mastrevirus*, *Begomovirus*) based upon genome organization, insect vector, and sequence homology [1,2]. Geminiviruses serve as a good source for regulatory elements, which can be used to drive transgene expression in plants. Promoters from *Cauliflower mosaic virus* (CaMV) [3], *Cotton leaf curl Multan* virus (CLCuMuV) [4], and *Strawberry vein banding virus* (SVBV) [5] display strong and consistent constitutive expression in transgenic plants. The large intergenic region (LIR) of the DNA-A component from the monopartite begomovirus genome contains plant *cis*-acting DNA regulatory elements and transcription factor binding sites (TFBs) required for the control of viral gene expression and replication [6]. Sequence and functional characterization of large intergenic regions (LIR) from monopartite and common regions from bipartite geminiviruses *Digitaria streak virus* (DSV) [7], *Tomato golden mosaic virus* (TGMV) [8,9,10,11], *African cassava mosaic virus* (ACMV) [12,13,14,15], *Maize streak virus* (MSV) [16], *Cotton leaf curl Multan* (CLCuMuV) [4], Wheat dwarf geminivirus (WDG) [17], *Mungbean yellow mosaic India virus* (MYMIV) [18], and Mungbean yellow mosaic geminivirus (MYMV) [19] revealed that this region, which is located between the 5' ends of the first complementary and virion sense open reading frames (ORFs), possesses promoter activity and is essential for the bidirectional transcription of both complementary (Rep) and virion (Cp) genes. In the case of ACMV, TGMV, CLCuMuV, and MYMIV, the LIR has much stronger promoter activity in the complementary sense than in the virion sense in the absence of transcriptional activator protein C2 [4,8,12,18,20]. 

Transcription of the replication associated protein (Rep) gene and coat protein (Cp) gene is governed by a bidirectional promoter that is present in the large intergenic region (LIR). Rep downregulates its own expression by binding to an iterative motif located between the TATA box and transcription start site [20]. The LIR also possesses an origin of replication (ori) for the viral genome. The stem-loop structure motif and iterated elements (8–13 nt) have been identified in the vicinity of the putative TATA box in the complementary (C1) sense promoter [21,22]. The iterated elements have been suggested to play pivotal roles in both the replication and transcriptional repression of complementary sense genes [22].

Cotton leaf curl Burewala virus (CLCuBuV) is a whitefly-transmitted monopartite begomovirus that infects cotton and has a recombinant genome composed of sequences derived from the *Cotton leaf curl Multan virus* (CLCuMuV) and *Cotton leaf curl Kokhran virus* (CLCuKoV) [23,24,25]. The DNA-A component of the monopartite begomovirus genome is organized into six open reading frames (ORFs), C1 (Rep), C2 (Trap), C3, C4, V1 (CP), and V2, which are transcribed bidirectionally from the LIR [6,26,27].

In this study, *Agrobacterium*-mediated delivery into plants was used as a transient assay system to qualitatively, as well as quantitatively study promoter activity. The LIR sequence from the CLCuBuV genome was isolated, and CLCuBuV C1and V1 promoter activity was investigated using GUS reporter gene transient expression in plants. We found that the CLCuBuV C1 promoter had strong and consistent transient expression in plant leaves compared to the CLCuBuV V1 and CaMV 35S promoters. The present study was designed to characterize the bidirectional gene promoter of CLCuBuV to use it in recombinant DNA technology to combat the spatio-temporal expression pattern of an insecticidal gene (*Cry1Ac*) in transgenic cotton in the future.

## 2. Experimental Section

### 2.1. Plant Material and Bacterial Strain

Seeds from a cotton (*G. hirsutum*) cultivar (MNH-786) were grown in composite soil (peat, sand, soil 1:1:1) in greenhouse at 25 + 2 °C. Seeds from tobacco (*N. tabacum*) were grown in loamy soil in a farmhouse at Chak No.59/F Hasilpur, Pakistan. After four weeks, the plants were moved greenhouse. Seeds from legumes (Mung bean, French bean, and Cow pea) were grown in Murashige and Skooge (MS) medium under a 16 h light and 8 h dark cycle at 22 °C. After one week, the older plants were subjected to transient expression. The *Escherichia coli* strain DH5α cells were used to clone all of the recombinant plasmid vectors. The *Agrobacterium tumefaciens* strain LBA4404 was used for the leaf and root infiltration. 

### 2.2. Isolation of CLCuBuV Bidirectional Promoter

Based on the characterized CLCuBuV genome, 455 bp fragments from both CLCuBuV C1 and V1 were amplified from a CLCuBuV genomic plasmid using promoter-specific primer sets. These primers were designed from the LIR of the CLCuBuV genomic clone (accession number FR750318) using the Primer 3 online software version 0.4 [28]. The PCR conditions were as follows; 94 °C for 3 min followed by 30 cycles of 94 °C for 45 s, 58 °C for 30 s, and 72 °C for 45 s, followed by a final extension at 72 °C for 5 min while the final holding temperature was 4 °C. The primer sets were as follows:

(i) 5'- CCATGGTGACTTTGGTTTAGAGACAACAAC-3' and 5'- CTGCAGTAATTCCTAGCCCTTATTACCAG-3'
(ii) 5'- CTGCAGTGACTTTGGTCAATTAGAGACAAC-3' and 5'- CCATGGTAATTCCTAGCCCTTATTACCAG-3'

The underlined sequences are the restriction enzyme sites engineered for cloning of both promoters. The contents of the 20 µL reaction PCR reaction were: 10 µL PCR master mix (Thermo Fisher Scientific, Waltham, MA, USA), forward primer 1 µL (10 µM), reverse primer 1 µL (10 µM), template 0.5 µL, and 7.5 µL of PCR grade DNase/RNase-free distilled water (Invitrogen, Carlsbad, CA, USA). The amplicons were separated on 1.5% agarose gel in Tris-Acetate-EDTA buffer, pH 8.0 and stained using ethidium bromide staining. Bands were visualized under UV on gel documentation system. The amplicons were cloned into an Invitrogen TA vector (pCR^®^2.1). 

### 2.3. Plasmid Construction

The binary vector pCAMBIA1301 (Cambia, Canberra, Australia) was used in the *Agrobacterium* transient plant transformation experiment. The T-DNA region of pCAMBIA1301 includes a selectable marker gene construct for hygB resistance and CaMV 35S promoter upstream of the GUS reporter gene. The CaMV 35S promoter was removed by excision of the *Nco* I–*Pst* I fragment containing the 35S promoter. The CLCuBuV C1 and V1 promoters were digested from the TA vector using the *Nco* I–*Pst* I restriction enzymes and subsequently cloned into pre-digested pCAMBIA1301 at the desired sites. The resulting plasmids were named pC1GUS1301 and pV1GUS1301. The integration of the promoters in both constructs was confirmed by restriction enzyme digestion and PCR. 

### 2.4. Sequence Analysis

The bidirectional promoter sequence was analyzed for the presence of *cis*-regulatory elements using the Plant *Cis*-Acting Regulatory Element (PlantCARE) database [29]. To identify the TFBs, we analyzed the activity of *cis*-elements using PLAnt *Cis*-acting Elements (PLACE) database [30]. The program SIGSCAN version 4.0.5 was used to search for more TFBs from the TRANScription FACtor database (TRANSFAC) database [31]. The transcription start site (TSS) was predicted using the Neural Network Promoter Prediction (NNPP) server [32]. Nucleotide sequence comparisons were performed using the CLUSTALW algorithm in the MegAlign program using the default settings in the Lasergene software from DNASTAR Inc. (Madison, WI, USA). The overrepresented CREs were identified using the MEME (Multiple EM for Motif Elicitation) tool version 4.4.0 [33]. The sequence logo was obtained from JAPSAR database [34].

### 2.5. Preparation of the *Agrobacterium* Suspension

Approximately 250 µL of the *A. tumefacience* strain of LBA4404 containing individual constructs was inoculated in 5 mL of YEP solution supplemented with kanamycin (50 µg/µL) and grown at 28 °C for two days while shaking at 180 rpm. Approximately 500 µL of *Agrobacteria* was then transferred to 50 mL of fresh YEP solution containing 10 mM MES, 20 µM acetosyringone and kanamycin at the same final concentration as above. After 24 h in culture (28 °C, 180 rpm), the *Agrobacterium* cells were collected by centrifugation for 10 min at 3,000 rpm and resuspended to an OD_600_ of 0.6–0.9 in suspension solution (MS medium supplemented with 10 mM MES and 200 mM acetosyringone) and incubated at room temperature for 2 h before Agroinfiltration.

### 2.6. *Agrobacterium*-Mediated Infiltration

*Agrobacterium*-mediated transient transformation of the tobacco and cotton leaves was conducted in middle-sized to near fully expanded leaves that were still attached to old transplanted plant seedlings. The experiment was repeated on three individual plants with five infiltrations each. Using a 1 mL syringe, 500 µL of the bacterial suspension was infiltrated into the intercellular spaces on the abaxial side of each intact leaf. The infiltrated borders were marked with a permanent marker. The plants were placed into a phytotron and analyzed after three days. Agroinoculation was carried out in the root-hypocotyl axis region of old sprouted seeds of Cowpea, French bean and Mung bean. After 72 h, the seedlings were trimmed, washed well to remove traces of the *Agrobacterium* culture on the surface and used for qualitative and quantitative assays of the reporter gene GUS. 

### 2.7. Histochemical Detection of GUS Activity

GUS activity was histochemically detected by incubating overnight at 37 °C in staining solution composed of 0.1% w/v 5-bromo-4-chloro-3-indolyl*-*β-d-glucoronic acid (X-Gluc; Sigma, St. Louis, MO, USA) in 100 mM Na_2_HPO_4_ pH 7.0, 0.01% w/v Chloramphenicol, 20% Triton X-100, 20% v/v Methanol. Chlorophyll was extracted from the photosynthetic tissues with 70% v/v ethanol. Transient GUS expression was detected microscopically by visualizing the distinct blue color that results from the enzymatic cleavage of X-Gluc. The samples were stored at 4 °C in 70% v/v ethanol.

### 2.8. Fluorometric Determination of GUS Activity

The infiltrated marked leaf and root areas were excised and frozen in liquid nitrogen. The frozen plant tissue was ground into a fine powder. After grinding, 1.0 mL of extraction buffer was mixed and centrifuged for 10 min (13,000 rpm) at 4 °C. A 5 µL aliquot of supernatant was mixed with 400 µL of pre-warmed (37 °C) GUS assay solution. The mixture was incubated at 37 °C, and 1.6 µL of the stop buffer (0.2 M Na_2_CO_3_) was added after 15 min. The stop buffer and 50 nM to 1 µM of 4-methylumbelliferone (4-MU) was used for calibration and standardization. The relative fluorescence was measured using a TKO 100 fluorometer (Hoefer Scientific Instruments, San Francisco, CA, USA) with an excitation wavelength of 360 nm and an emission wavelength of 465 nm. The detected fluorescence was expressed in relative fluorescence units (RFU) (1pM-4MU is equal to 1RFU mg^−1^ protein min^−1^). The protein concentrations were estimated by performing the Bradford assay [35]: 20 μL extract was mixed with 780 μL 1× PBS buffer and added to 200 μL dye reagent concentrate (Bio-Rad, München, Germany). Sterile water (dye reagent added) was used for reference setting and 10 mg/mL to 100 mg/mL bovine serum albumin (Sigma, Steinheim, Germany) (dye reagent added) were used as standards. Protein concentration in the extract was determined using a standards-based calibration curve (exponential) established with Microsoft Excel.

### 2.9. Statistical Analysis

The quantification expression data was analyzed statistically by using ANOVA and Tukey’s HSD (honest significant difference) and Fisher’s LSD (least significant difference) (individual and pairwise) for completely randomized designs (CRD) by using a Statistix 8.1 software. *p* value of <0.05 was considered as statistically significant.

## 3. Results

### 3.1. Structure and Sequence Analysis

**Figure 1:**
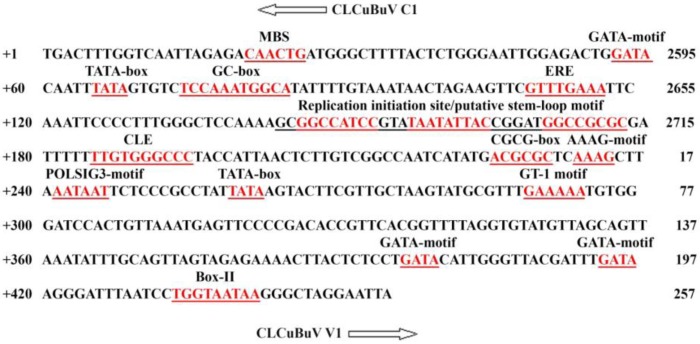
The 455 bp DNA sequence of the full-length transcript of the bidirectional gene promoter of the CLCuBuV coordinates from 2595–292 nucleotides in the viral genome. The putative motifs are in bold and underlined. The replication initiation site/putative stem-loop motif was composed of 35 bp consisting of ATC-motif (GGCCATCC) and GC-rich region (GGCCGCGC) around the nonanucleotide. The conserved late element (CLE) was present at position +185 was suggested to functional target for C2. The POLASIG3 motif (AATAAT) was also identified at position +242. The TATA-box for Rep promoter was found at position 65 nucleotide and TATA-box for Cp promoter was located at position 259 nucleotide in the LIR. The Box-II are predicted at position 433 in the LIR. MBS (MYB drought responsive element) was found at position 20 within the LIR. The labeling of motifs are indicated above the line. The right-side numbering shows the position of LIR in the genome.

The CLCuBuV bidirectional gene promoters were isolated from the LIR of the DNA-A genomic clone from CLCuBuV, as previously characterized [25]. The complete DNA-A component of the CLCuBuV genome has been reported (Genbank accession number: FR750318 CLCuBuV-MV12). The nucleotide sequence of the CLCuBuV bidirectional gene promoter (CLCuBuV genomic coordinates 2595–292 bp) is shown in (Figure 1). 

**Figure 2:**
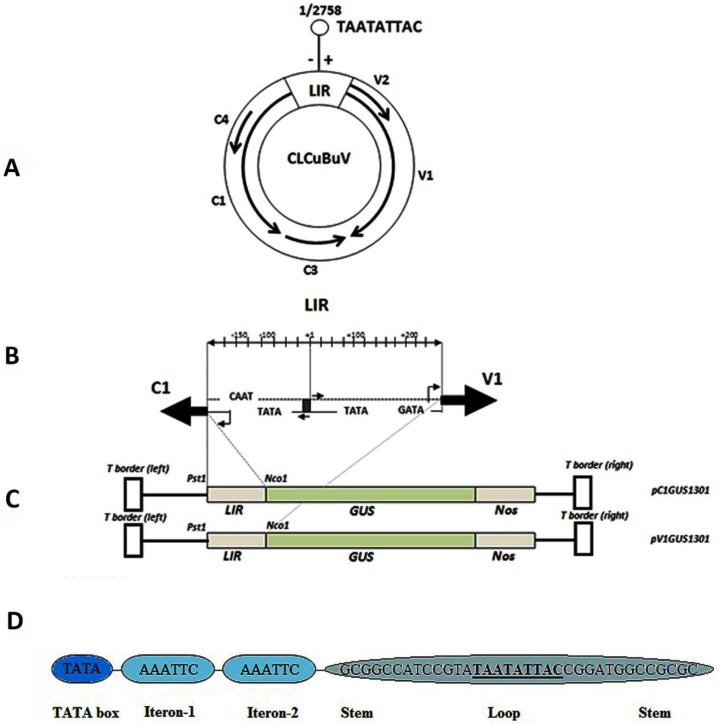
(**A**) Genome organization of the DNA-A component of CLCuBuV. Five ORFs are shown with the arrows. V1 and V2 (coat protein) are located on the plus strand, C1 (replication associated protein), C3 (replication enhancer protein) and C4 (unnamed) are located on the complementary sense strand. The 455 bp large intergenic region (LIR) contains the potential stem-loop with nonanucleotides conserved among all geminiviruses. Sequence numbering begins at base 8 of the conserved nonamer; (**B**) A schematic drawing of the CLCuBuV LIR. Shown, is the invariant loop sequence with the nonanucleotide sequence (grey box) flanked by inverted repeats (short arrows); the viral protein start sites and replication associated proteins; (**C**) A schematic representation of both expression vectors shows the cloning of the C1 and V1 promoters; (**D**) Schematic representation of important features of large intergenic region (LIR) of CLCuBuV including REP-binding sites (iterons), TATA box and structure of hair pin showing nonanucleotide (underlined). The TATA box for Rep promoter was to be present at position 2660–2664 in the genome. Two directly repeats iteron motifs AAATTC were predicted at position 2709–2715; 2715–2721 present downstream to TATA box and immediate upstream to stem-loop motif .The stem-loop motif composed of 35 bp was predicted at position 2738–15 in the CLCuBuV genome.

The cloning of both promoters is represented in Figure 2. The bidirectional promoter sequence contains several consensus eukaryotic regulatory domains, such as TATA, GC-rich, and CAAT boxes, which are present in almost all geminivirus LIRs [21,36]. The LIR consisted of 455 bp and contained *cis*-acting DNA elements that are involved in begomovirus replication and transcriptional regulation, including a Rep-binding site (iterons), TATA boxes, GC boxes, and stem-loop elements that contain the conserved nonanucleotide (TAATATTAC) sequence. The TATA box is located 30 bp upstream of the transcription start site. The TATA boxes and GC-rich box have been shown to be essential for geminivirus promoter activity within the LIR. The putative CLE (TTGTGGGCCC) was suggested to be a potential functional target for C2 to trans-activate virion sense gene transcription [21,37]. The sequence comparison revealed several potential TFBs in the CLCuBuV LIR region, such as the E-box motifs (CANNTG) [38] at sites +21, +77, +220, −21, −77, and −220 and the maize DOF transcription factor recognition core sequence AAAG [39] at sites +141, +234, −4, −32, and −129. The TATA and CAAT boxes are located at +141, +234, −4, −32, and −129. The CARGCW8GAT (CWWWWWWWWG) motif, which are responsible for regulating nectory-specific gene expression, at positions +61, +256, −61, and −256. The plant polyA signal consensus sequence POLASIG3 motif (AATAAT) was also identified at position +242. Other potential plant *cis*-regulatory DNA elements and TFBs that were identified searching the PlantCARE and PLACE databases are listed in Table 1 and Table 2. 

**Table 1:** List of putative *cis*-regulatory elements found in the large intergenic region (LIR) of CLCuBuV.

No.	TFBs ^a^	Function of the motif	organism	Sequence	Strand	Position
1	Box-I	LRE ^b^	*Pisum sativum*	TTTCAAA	−	110
				TTTCAAA	−	286
2	Box-II	LRE	*Solanum tubersum*	TGGTAATAA	+	433
3	Box-W1	Fungal elicitor RE	*Petroselinum crispum*	TTGACC	−	7
4	C-Repeat/DRE	Cold and dehydration RE	*Arabidopsis thaliana*	TGGCCCGAC	−	209
5	CAAT-box	Core promoter elements	many	many	+,−	many
6	CGCG-box	Signal RE	*Arabidopsis thaliana*	ACGCGC	+	226
				GCGCGG	−	173
7	CGTCA-motif	MeJA RE	*Hordeum vulgare*	CGTCA	−	223
8	Circadian	Circadian control	*Lycopersicon esculentum*	CAANNNNATC	−	178
9	ERE	Ethylene RE	*Dianthus caryophyllus*	ATTTCAAA	+	110
10	GATA-motif	LRE	*Arabidopsis thaliana*	GATA	+	many
11	G-box	LRE	*Triticum aestivum*	TCCACATGGCA	+	74
12	GC-motif	unknown	*Oryza sativa*	GCCGCGCCG	+	171
13	GT-1motif	LRE	*Avena sativa*	GGTAAT	−	161
			*Oryza sativa*	GAAAAA	+	290
			*Pisum sativum*	GGTAAT	+	435
14	HSE	HSE ^c^	*Brassica oleracea*	AGAAAACTT	+	380
15	I-box	LRE	*many*	many	+,−	many
16	MBS	MYB drought RE	*Arabidopsis thaliana*	CAACTG	+	20
				TAACTG	−	355
				TAACTG	−	366
17	Skn-1_motif	Endosperm expression RE	*Oryza sativa*	GTCAT	−	222
18	TATA-box	Core elements located at −30 of TSS	many	many	+,−	many
19	TGACG-motif	MeJA RE	*Horedeum volgare*	TGACG	+	223
20	W-box	LRE	*Arabidopsis thaliana*	TTGACC	−	7

Both the AC1 and AV1 promoters share common promoter elements. The LIR is flanked by the Rep and virion sense genes. The nucleotide immediately preceding the Rep (C1) gene is numbered 1; ^a^ Transcriptional factor binding sites; ^b^ Light responsive elements; ^c^ Heat shock responsive elements.

**Table 2:** The putative transcriptional factor binding sites (TFBs) identified from the PLACE database.

TFBs	PLACE ID	PLACE accession ID	Sequence	Copy number	Description
Cytokinin related	ARFAT(Aux RE)	S000270	NGATT	2	“ARR1-binding element” found in rice andArabidopsis;ARR1 is response regulator;N = G/A/C/T [40]
	ARR1AT	S000454	TGTCTC	5	“ARF biding site” found in the promoters of primary/early response gene [41]
Auxin related	SURECOREATSULTR11	S000499	GAGAC	8	Core of sulfur-responsive element SURE); containing ARF binding sequence GAGACA(complementary AuxRE TGTCTC)[42]
	CATATGGMSAUR	S000370	CATATG	2	Multiple auxin response modules in the soybean SAUR15A promoter [43]
Mesophyll-specific	CACTFTPPCA1	S000449	YACT	8	Mesophyll-specific gene expression in C4 plant Flaveria trinervia [44].
Pollen-specific	POLLEN1LELAT52	S000245	AGAAA	1	Pollen-specific expression of tomato Late52 gene [45]
Root-specific	ROOTMOTIFTAPOX1	S000098	ATATT	5	Root-specific motifs found in the rolD promoter [46].
	OSE2ROOTNODULE	S000468	CTCTT	1	Nodule specificity of soybean lbc3 and N23 gene promoters [47].

### 3.2. Transient Expression of the Bidirectional Promoter

**Figure 3:**
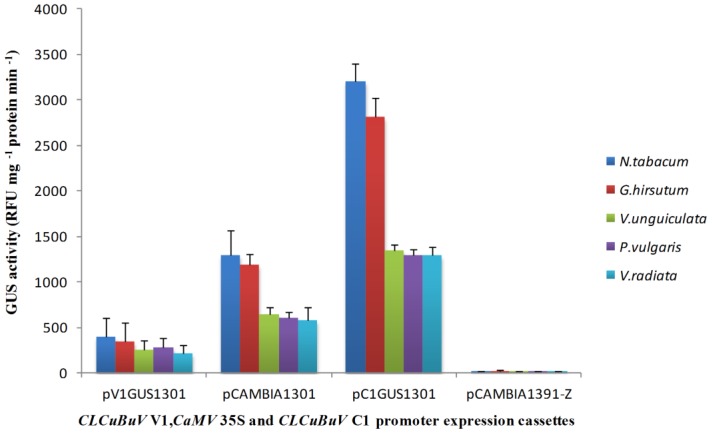
Transient GUS expression in plant leaves and roots agro-inoculated with the AC1 and AV1 promoter constructs from CLCuBuV (pV1GUS1301, pC1GUS1301) in comparison with positive control CaM 35S promoter (pCAMBIA1301) and a negative promoterless control (pCAMBIA1391-Z). Error bars represent standard deviation.GUS activity measured in RFU mg^−1^protein min^−1^. Each treatment was carried out in three replications.

In the agroinfiltration of the leaves in both hosts (cotton and tobacco), the AC1 promoter construct (pRepGUS1301) revealed Gus-positive expression in vascular and mesophyll cells. The AV1 promoter construct (pCPGUS1301) showed very weak GUS transient expression. The CaMV 35S promoter also displayed high GUS activity levels. There was no difference in the pattern of expression of the AV1 and AC1 promoters at 48 and 72 h post-delivery into the plants. To quantify GUS expression, two separate leaves of *N. tobacum* were agroinoculated with each promoter construct in triplicate. Equivalent amounts of protein were harvested and assayed for fluorescence. The CLCuBuVC1 promoter showed higher activity when compared with the CLCuBuV V1 and CaMV 35S promoters, as shown in (Figure 3) GUS activity from each promoter construct was highly uniform in the different leaves. Transient GUS expression from all three constructs was uniform in both host plant leaves. All three constructs were GUS positive in the root hypocotyl axis of the sprouted French bean seeds. The transient expression of the CLCuBuV V1 promoter was very low compared to the CLCuBuV C1 promoter; one or two blue spots were observed microscopically in few explants. To determine whether the CLCuBuVC1 promoter was differentially expressed in other legume hosts, cow pea, and mung bean seedlings were also tested. All three constructs had similar GUS expression levels in these hosts as in the French bean seedlings as shown in (Figure 3). The statistical analysis revealed that there were significant differences (*p* < 0.05) for all promoters. Lowest coefficient of variance was recorded for cow pea while higher for French bean. Highest mean performance was recorded for tobacco (1,320 ± 441.99) while lowest for French bean (532.72 ± 247.45). The results indicated that CLCuBuV C1 promoter performed better as compared to other promoters in all plants while lower performance was recorded for CLCuBuV V1 promoter. The combined effects of all promoters indicated significant differences among the promoters, crops, and their attractions. 

### 3.3. Nucleotide Sequence Comparison of the CLCuBuV LIR

The 455 bp LIR sequence of CLCuBuV acts as a bidirectional promoter and is homologous to the LIRs from several other CLCuV isolates. The CLCuBuV LIR showed 81%–82% nucleotide sequence identity with the LIR from CLCukoV, CLCuMuV, and Cotton leaf curl Shahdadpur virus (CLCuShV) but only 32% and 65.7% sequence identity with the LIRs from *Cotton leaf curl Gezira virus* (CLCuGeV) and *Cotton leaf curl Alabad virus* (CLCuAV), respectively. The nucleotide sequence identities of the CLCuBuV LIR with other begomoviruses are listed in (Table 3). The transcription start site was predicted to be 30 bp downstream of the consensus TATA box. This is consistent with the PlantCARE database results, which indicate that the consensus TATA box is located at the −30 site of TSS, as listed in Table 1. These results are in agreement with a previous study involving the transcript mapping of CLCuBuV [48]. The A+T content of the CLCuBuV LIR is approximately 60%. The CLCuBuV bidirectional gene promoter showed 70% nucleotide sequence identity with the WDG [17]. 

**Table 3:** Nucleotide sequence comparison of the LIR from CLCuBuV with other closely related geminivirus LIRs.

No	Virus Acronym	% identity ^a^	LIR size (bp)	Accession IDs
1	*CLCuBuV*	100	455	FR837932
2	*CLCuMuV*	81.8	440	AJ496287
3	*CLCuKoV*	81.6	447	AJ496286
4	*CLCuShV*	81.9	447	FN552004
5	*HYVMV*	77.4	437	FR772082
6	*CLCuBaV*	76.4	444	NC_007290
7	*MaYVCMV*	73.6	440	FR715681
8	*PaLCuV*	70.1	478	FM955602
9	*AEV*	69.9	445	AM698011
10	*CYVMV*	69.8	451	FN645926
11	*ToLCPKV*	68.1	456	AM948961
12	*ChiLCMuV*	68.7	450	FM149613
13	*SiLCV*	66.9	447	DQ641706
14	*CLCuAaV*	65.2	434	AJ002452
15	*CLCuRaV*	64.6	444	JF502364
16	*CLCuGeV-PK*	32.7	453	FR751142
17	*CLCuGeV-SD*	32.6	451	AY036007

^a^ % identity with CLCuBuV LIR.

### 3.4. Enriched Regulatory Elements in the Geminivirus Bidirectional Promoters

**Figure 4:**
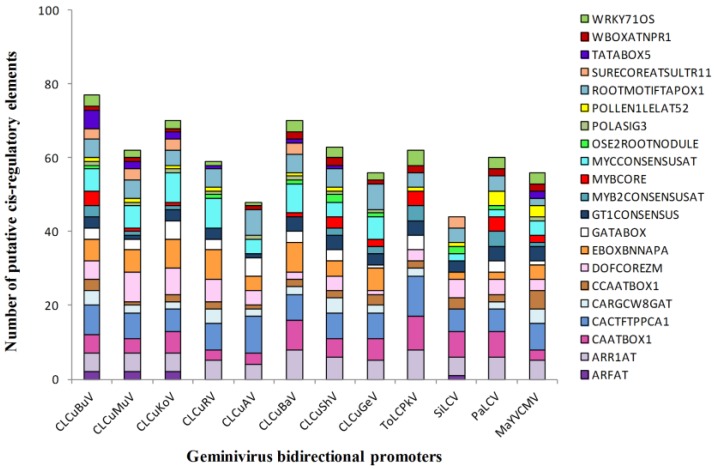
Occurrence of 21 enriched *cis*-acting regulatory DNA elements distributed in the 12 bidirectional geminivirus promoters.

Transgene expression is regulated by the interaction of transcription factors and target *cis*-regulatory elements in the promoters. The identification of potential regulatory elements acting in the geminivirus bidirectional promoters can be a useful tool to understand the regulatory network. We scanned enriched *cis*-acting regulatory DNA elements in the bidirectional promoters of CLCuBuV and closely related geminiviruses to determine possible functions. We first analyzed known plant regulatory elements in these promoter sequences using PLACE collection. We used CREs within the LIR of CLCuBuV and other geminivirus to compare different sequences. The names of 21 broadly distributed CREs and their occurrence in each promoter sequence are shown in Figure 4.

**Figure 5:**
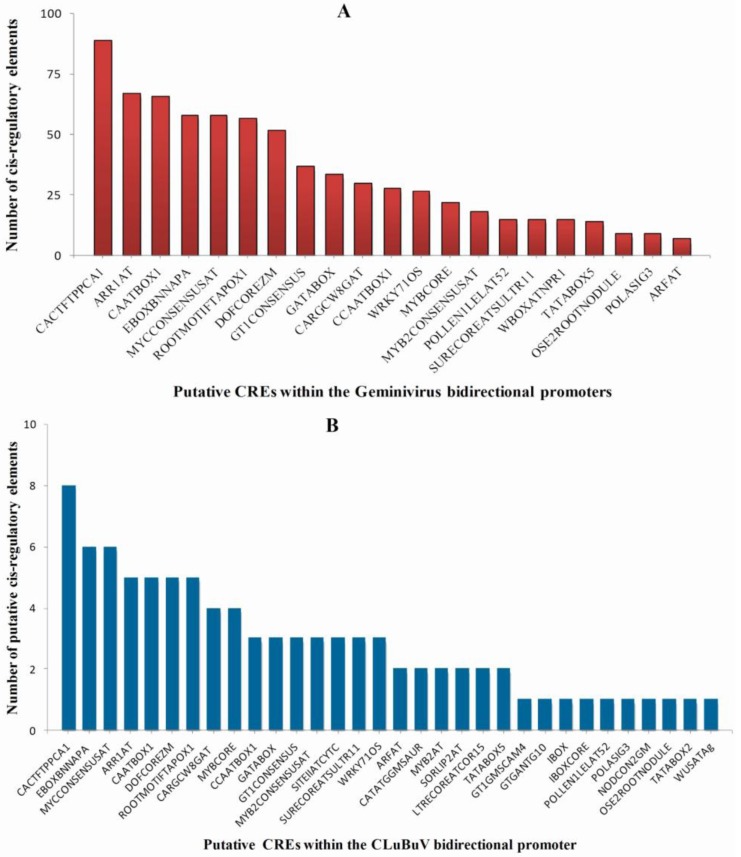
(**A**) Occurrence of the top 21 CREs with a total occurrence in 12 promoters; (**B**) Occurrence of 32 CREs distributed in the CLCuBuV bidirectional promoter.

**Figure 6:**
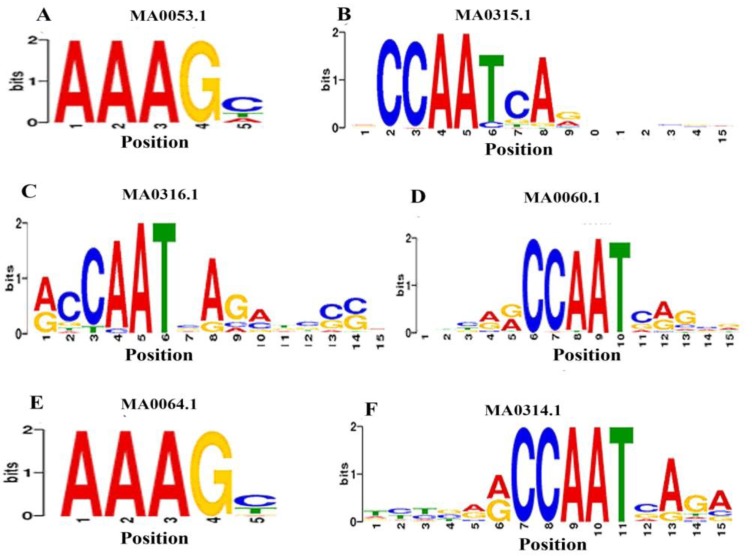
Overrepresented sequences in the bidirectional promoters of geminiviruses used in this study. Letters abbreviating the nucleotides (A, C, G, T) in the images are sized to their relative occurrence. (**A**) MNB1A motif (MA0053.1) *p*-value 3.1e-075 belongs to Zinc-coordinating class and Dof family; (**B**) HAP4 motif (MA0315.1) *p*-value 4.2e-027; (**C**) HAP5 motif (MA0316.1) *p*-value 7.33e-05; (**D**) NFYA motif (MA0060.1) *p*-value 5.3e-06; (**E**) PBF motif (MA0064.1) *p*-value 1.4e-017 belongs to Zinc-coordinating class and Dof family; (**F**) HAP3 motif (MA 00314.1) *p*-value 1.2e-07. All HAP motifs belong to Alpha Helix class and NFY CCAAT-binding family.

The ranking of these 21 CREs with total occurrence are shown in Figure 5A. The distribution and occurrence of the CREs in the bidirectional promoter of CLCuBuV is shown in Figure 5B. Several enriched PLACE motifs are universal or structural CREs that are similar to geminivirus bidirectional promoters, such as TATABOX5 [49], POLASIG1, and POLASIG3 [50,51]. Many CREs are environmental responsive motifs, such as MYCCONSENSUSAT for cold [52,53], WRKY71OS for gibberellins and pathogenesis [54,55], IBOXCORE for lights [56,57], MYBCORE for water stress [58], and GT1GMSCAM4 for pathogens and salt [59]. To complement the searches for possible motifs that have a statistically overrepresented frequency in the LIR, we computationally analyzed the closely related promoters using MEME. As a result, six motifs were found enriched within the twelve promoters (Figure 6).

### 3.5. Identification of TFBs that Bind to the Promoters of ds-DNA Animal Viruses

The TFBs in various organisms were searched for in the databases to determine more regulatory elements in the large intergenic region of CLCuBuV. Apart from the sites for TFs that bind to promoter elements, more than 40 TFBs were identified in plants, mammals and yeast. The binding sites of TFs that bind to the promoters of certain ds-DNA animal viruses were also found, such as CRF and NF-1, which bind to the E2 late promoter [60] and early gene (E1A) [61], respectively, of adenoviruses; Cx and NF-Y, which bind to immediate early gene-3 (IE-3) [62] and thymidine kinase [63], respectively, of herpes simplex virus (HSV-1); and T-Ag that binds to Polyoma viruses [64].

## 4. Discussion

*Agrobacterium*-mediated transient transformation has been used to analyze foreign gene expression [65,66,67,68], gene silencing [69,70], and gene interactions [71,72,73]. More recently, the *Agrobacterium*-mediated transient expression assay has been demonstrated to be a simple and efficient method for the quantitative analysis of plant promoter and *cis*-element/trans-factor interactions *in vivo* [74]. In a previous study, our group has characterized the cotton universal stress protein promoter and cotton heat shock protein promoter in response to abiotic stresses in tobacco using the *Agrobacterium*-mediated transient assay [75,76]. We utilized six-week old tobacco leaves for agro-infiltration because of the excellent transformation efficiency and minimization of assay variation [74].

In this study, the LIR sequence of the DNA-A component of the CLCuBuV genome was isolated and analyzed. In the transient expression assay with tobacco and cotton leaves, the CLCuBuV C1 promoter displayed strong GUS activity, while the CLCuBuV V1 promoter displayed weak activity in the absence of the C2 (Trap) gene product. CLCuBuV lacks the transcriptional activator protein C2 (Trap) [23,25]. The GUS quantitative assay revealed that CLCuBuV C1 promoter activity was two- to three-fold higher than the CaMV 35S promoter, while the activity of CLCuBuV V1 was three- to four-fold lower than the CaMV 35S promoter. It has been reported that virion sense promoters need trans-activator AC2/C2 [77,78]. The superiority of the C1/AC1 promoter compared to the V1/AV1 promoter in the absence of transcriptional activator protein C2 (Trap) was also reported in ACMV [12], wheat dwarf virus (WDV) [79], CLCuMuV [4], and MYMIY [18]. We have previously characterized the DNA-A component of the CLCuBuV genome and phylogenetically analyzed the LIR, which displayed homology with the LIRs from CLCuKoV and CLCuMuV [25]. The transient GUS expression analysis of CLCuBuV C1 and V1 in this study is consistent with the CLCuMuV C1 promoter, which displayed four- to five-fold higher GUS activity than the CaMV 35S promoter in transgenic tobacco plants [4]. Similar to other geminiviruses, CLCuBuV produces multiple overlapping polycistronic RNA species that diverge from the LIR, confirming a bidirectional transcriptional strategy. The TATA and CAAT boxes are located approximately 30 bp from the transcription initiation site (TIS), which is consistent with a previous study involving the long complementary sense transcript mapping of CLCuBuV [48]. This previous study suggested that CLCuBuV uses a bicistronic transcription strategy to translate C2 and REn (replication enhancer protein) from a single transcript [48,80]. The homologous regulatory modules responsive to light, heat, wounds, hormones, and salicylic acid that have been identified in different monocots and dicots were found in the bidirectional promoter of CLCuBuV. The transcription regulation of geminiviruses share many common features with papavoviruses and adenoviruses. In that context the presence of TF binding sites in the CLCuBuV bidirectional promoter that are also found in adenoviruses and papavoviruses gain significance. Though hundreds of begomoviruses have been cloned and sequenced, analysis of promoters had been carried out for only few begomoviruses. The information that we have generated on the bidirectional promoter of CLCuBuV, a very distinct newly emerged Old World begomovirus would help in further elucidating transcription regulation in begomoviruses. The expression of RNAi construct (hairpin) of LIR of CLCuBuV to control Cotton leaf curl disease (CLCuD) and expression of an insecticidal gene (Cry1Ac) under the CLCuBuV C1 promoter in transgenic cotton are in progress.

## 5. Conclusions

In conclusion, we have computationally characterized the bidirectional gene promoter from CLCuBuV and 11 other closely related geminiviruses that specifically drive gene expression. These findings provide important tools for transgene expression studies and crop breeding. Particularly, of the promoters studied, CLCuBuV C1 is a prime candidate for high gene expression, which is desired in green tissues, using recombinant DNA technology. 
